# Supplementary material for: Role and mechanism of NCAPD3 in promoting malignant behaviors in gastric cancer
Source: Front Pharmacol. 2024 Apr 22;15:1341039. doi: 10.3389/fphar.2024.1341039 (PMC11070777; doi:10.3389/fphar.2024.1341039)
Supplement: Supplementary file 11 [file DataSheet2.ZIP › GSEA/Oncogenic signature/my_analysis.Gsea.1599461962066/CYCLIN_D1_UP.V1_DN.html]

Details for gene set CYCLIN\_D1\_UP.V1\_DN[GSEA]

|  || Dataset | filtered\_dataset.sample\_info.cls#WT\_versus\_NCAPD3\_MUT |
| Phenotype | sample\_info.cls#WT\_versus\_NCAPD3\_MUT |
| Upregulated in class | WT |
| GeneSet | CYCLIN\_D1\_UP.V1\_DN |
| Enrichment Score (ES) | 0.1492956 |
| Normalized Enrichment Score (NES) | 0.67316854 |
| Nominal p-value | 0.858885 |
| FDR q-value | 1.0 |
| FWER p-Value | 1.0 |
Table: GSEA Results Summary

  

Fig 1: Enrichment plot: CYCLIN\_D1\_UP.V1\_DN      
 Profile of the Running ES Score & Positions of GeneSet Members on the Rank Ordered List

  

| SYMBOL | TITLE | RANK IN GENE LIST | RANK METRIC SCORE | RUNNING ES | CORE ENRICHMENT || 1 | 2072 | ERCC4 | 7 | 1.130 | 0.0808 | Yes |
| 2 | 7170 | TPM3 | 24 | 1.018 | 0.1467 | Yes |
| 3 | 7024 | TFCP2 | 204 | 0.671 | 0.0686 | Yes |
| 4 | 4238 | MFAP3 | 279 | 0.609 | 0.0615 | Yes |
| 5 | 9674 | KIAA0040 | 315 | 0.586 | 0.0808 | Yes |
| 6 | 5465 | PPARA | 375 | 0.550 | 0.0801 | Yes |
| 7 | 2997 | GYS1 | 376 | 0.548 | 0.1217 | Yes |
| 8 | 22796 | COG2 | 445 | 0.507 | 0.1112 | Yes |
| 9 | 5295 | PIK3R1 | 467 | 0.493 | 0.1336 | Yes |
| 10 | 3479 | IGF1 | 511 | 0.469 | 0.1382 | Yes |
| 11 | 3609 | ILF3 | 544 | 0.449 | 0.1493 | Yes |
| 12 | 56252 | YLPM1 | 665 | 0.394 | 0.0927 | No |
| 13 | 5134 | PDCD2 | 678 | 0.388 | 0.1136 | No |
| 14 | 23212 | RRS1 | 681 | 0.387 | 0.1416 | No |
| 15 | 694 | BTG1 | 901 | -0.326 | 0.0085 | No |
| 16 | 687 | KLF9 | 996 | -0.402 | -0.0288 | No |
| 17 | 7277 | TUBA4A | 997 | -0.404 | 0.0019 | No |
| 18 | 901 | CCNG2 | 998 | -0.405 | 0.0327 | No |
| 19 | 2146 | EZH2 | 1001 | -0.406 | 0.0621 | No |
| 20 | 857 | CAV1 | 1098 | -0.475 | 0.0290 | No |
| 21 | 2825 | GPR1 | 1171 | -0.538 | 0.0180 | No |
| 22 | 6038 | RNASE4 | 1194 | -0.570 | 0.0454 | No |
| 23 | 6690 | SPINK1 | 1200 | -0.577 | 0.0856 | No |
| 24 | 4734 | NEDD4 | 1369 | -0.856 | 0.0296 | No |
Table: GSEA details [plain text format]

  

Fig 2: CYCLIN\_D1\_UP.V1\_DN      
 Blue-Pink O' Gram in the Space of the Analyzed GeneSet

  

Fig 3: CYCLIN\_D1\_UP.V1\_DN: Random ES distribution      
 Gene set null distribution of ES for **CYCLIN\_D1\_UP.V1\_DN**
